# Service-oriented cloud manufacturing systems: Balancing profit, customer satisfaction, and resource fairness

**DOI:** 10.1371/journal.pone.0343430

**Published:** 2026-07-23

**Authors:** Asra Moslemipour, Ali Salmasnia, Hadi Mokhtari

**Affiliations:** 1 Department of Industrial Engineering, Faculty of Engineering, University of Qom, Qom, Iran; 2 Department of Industrial Engineering, Faculty of Engineering, University of Kashan, Kashan, Iran; NOVA School of Science and Technology: Universidade Nova de Lisboa Faculdade de Ciencias e Tecnologia, PORTUGAL

## Abstract

The rapid growth of customized demand and geographically distributed manufacturing resources has increased the need for integrated decision-making in cloud manufacturing systems. In such environments, scheduling, logistics, pricing, and quality decisions are highly interrelated and significantly affect both system profitability and customer satisfaction. However, most existing studies address these decisions separately and pay limited attention to customer satisfaction and fairness. To address this gap, this paper proposes a multi-objective mixed-integer programming model that simultaneously integrates scheduling, logistics, pricing, and quality decisions while explicitly considering customer satisfaction and fairness among customers. The model pursues three objectives: maximizing cloud manufacturing system profit, maximizing customer satisfaction as a function of price and product quality, and minimizing unfairness among customers. An LP-metric approach is employed to aggregate the objectives, and the model is solved using CPLEX in GAMS. Computational experiments on small-, medium-, and large-scale instances demonstrate the effectiveness of the proposed framework. The results show that ignoring logistics decisions, customer satisfaction, or earliness/tardiness penalties leads to inferior solutions. Furthermore, a Genetic Algorithm is developed for large-scale instances, where the exact approach becomes computationally demanding. Comparative results indicate that the proposed heuristic provides high-quality solutions with significantly lower computational times for large-scale problems. The findings confirm the effectiveness and scalability of the proposed framework for integrated decision-making in cloud manufacturing systems.

## 1. Introduction

Customer and user needs and requirements in today’s competitive environment are changing rapidly, and many customers prefer to receive customized products with the best price and quality in the shortest possible time. While responding to such individualized demands is difficult for each company independently, manufacturing firms face serious challenges in coping with these changes. One of the major challenges is the geographical dispersion of manufacturing resources, the integration of which has always been accompanied by new obstacles. Fortunately, recent advances in information technology and the Internet of Things have made the path toward globalization more attainable. These advances enable companies to share various resources and technologies and, when necessary, to utilize external resources to address their production shortages. In this context, cloud manufacturing, as a service-oriented manufacturing model, facilitates the integration of manufacturing resources located in different geographical areas in such a way that customer demand can be satisfied with the best possible specifications Uncited. The concept of cloud manufacturing was first introduced by Li et al. [1] in their paper entitled *“Cloud Manufacturing: A Service-Oriented Networked Manufacturing Model.”*A cloud manufacturing platform is capable of performing multiple tasks simultaneously, and customers can submit their orders through this system. In cloud manufacturing systems, the term “task” is considered equivalent to an “order” or a “job.” After a task is submitted, the cloud manufacturing platform decomposes it into subtasks and determines the task structure; it then proceeds to schedule the subtasks. One of the key aspects of management in cloud manufacturing is the focus on subtask scheduling, during which limited resources are allocated to activities over time in such a way that one or more predefined objectives are optimized. In general, the purpose of scheduling is to determine the sequence in which subtasks are processed by service providers, in accordance with the product request time, so that products are delivered to customers on time.

Over the past decade, direct sales have become increasingly prevalent due to technological advances, particularly the development of the Internet. In general, this sales model facilitates the observation of, and even influence on, end-customer behavior. Under such conditions, issues related to pricing and production scheduling in manufacturing plants emerge and attract growing attention. Offering superior products compared to competitors and adopting appropriate strategies to achieve a favorable competitive position are fundamental principles for attaining profitability in business models. Accordingly, a manufacturing firm must be able to price its products in a way that generates revenue commensurate with the value delivered to customers, thereby maintaining its position vis-à-vis customers and competitors. An important point is that pricing and scheduling in cloud manufacturing systems are inherently interrelated. Therefore, they should be considered simultaneously in order to lead to more optimal decisions that ensure the survival and success of such systems. By integrating pricing and scheduling decisions, these systems can more effectively manage resources and profitability through the coordinated planning of production and order delivery.

In this context, considering logistics and selecting appropriate logistics services are crucial to ensuring the timely delivery of products to customers. Since factories are located in different geographical regions, they require inter-factory logistics services when collaborating to complete a manufacturing task. Finished products and components are delivered to service requesters through logistics networks by selected logistics service providers [2]. In service-oriented manufacturing models, manufacturing resources across different firms are integrated through a network consisting of cloud platforms and logistics systems. Therefore, establishing an efficient logistics service capable of providing reliable transportation throughout the execution and delivery of subtasks is essential for cloud manufacturing systems. In cloud manufacturing systems, customers and service providers each have different needs and expectations. On the one hand, customers seek to receive their orders at the lowest possible cost and with the highest attainable quality. On the other hand, the cloud manufacturing system must strive to maintain its profitability and operational efficiency. Under such conditions, designing a comprehensive model that can simultaneously satisfy the interests of both parties is of particular importance. Such a model can enhance customer satisfaction while preserving system profitability by establishing a balance among multiple objectives. Ultimately, this approach contributes to the long-term sustainability and efficiency of the entire cloud manufacturing system. In achieving this balance between satisfaction and profitability, fairness in the allocation of resources and services plays a decisive role. In cloud manufacturing environments, which involve two distinct groups, customers and service providers, ensuring fairness in the distribution of resources and services is of special significance. Establishing fairness not only increases the satisfaction of both parties but also represents a key factor in guaranteeing the long-term sustainability and development of these systems. The importance of this principle is such that it is consistently regarded as a fundamental criterion in the formulation of scheduling problems. Accordingly, the present study seeks to establish a balance between customer expectations and the operational objectives of the system by adopting fairness-based criteria in scheduling.

In the context of this study, the term “customer” refers to industrial service requesters or enterprise stakeholders that submit manufacturing tasks to the cloud manufacturing platform, rather than individual end-users or retail consumers. These customers are assumed to possess clear operational preferences regarding production cost, delivery performance, and product quality. Accordingly, customer satisfaction in this research is modeled based on quantifiable operational criteria, including pricing and product quality, which can be directly incorporated into the mathematical optimization framework.

In cloud manufacturing systems, most existing studies have focused solely on one perspective, either the cloud manufacturing system or the customer, by optimizing system costs while neglecting the interests of the other party. This approach constitutes a fundamental gap in cloud manufacturing research. In contrast, the objective of the present study is to simultaneously maximize the satisfaction of both the cloud manufacturing system and customers, with an emphasis on preserving the interests of both sides.

Based on prior studies and the identified research gaps in the field of cloud manufacturing systems, the main objective of this paper is to address the following two major challenges:

1. The lack of an integrated model for simultaneous decision-making regarding logistics operation scheduling, pricing, and quality, and for examining the impact of these decisions on customer satisfaction and fairness among customers.
2. The absence of a comprehensive framework capable of simultaneously optimizing multiple objectives, including maximizing the profit of the cloud manufacturing system, maximizing customer satisfaction, and minimizing unfairness among customers.

To address these research gaps, this study develops an integrated mathematical programming model that simultaneously considers decisions related to logistics operation scheduling, pricing, and quality, and examines the effects of these decisions on customer satisfaction and fairness among customers. In designing this model, a multi-objective approach is employed, pursuing three main objectives: maximizing the profit of the cloud manufacturing system, maximizing customer satisfaction, and minimizing unfairness in resource allocation among customers.

The main contributions of this research can be summarized as follows:

1. Proposing a comprehensive integrated model that simultaneously considers five major dimensions in cloud manufacturing systems, namely logistics operation scheduling, pricing, quality, customer satisfaction, and fairness among customers, which have rarely been addressed jointly in previous studies.
2. Modeling the simultaneous effects of price and quality on customer satisfaction.

To solve the proposed model, a multi-objective programming framework implemented in the GAMS software environment is employed. By simultaneously optimizing the three main objectives, the model provides an optimal solution that establishes an appropriate balance among the interests of the cloud manufacturing system, customer requirements, and the principle of fairness in resource allocation.

Section 2 provides a comprehensive review of the relevant literature and prior research. Section 3 describes the research problem, objectives, and the fundamental assumptions of the model. Section 4 presents the mathematical formulation of the proposed model. Section 5 is devoted to the analysis of numerical results, in which the proposed model is validated by solving small- and large-scale problem instances using the CPLEX solver within the GAMS environment; subsequently, its performance is evaluated through a comparative study with other mixed-integer programming models. Section 6 conducts a systematic sensitivity analysis to identify key parameters and derive managerial insights. Finally, Section 7 presents the summary of findings, conclusions, and directions for future research.

## 2. Literature review

Driven by market demand, the manufacturing industry has undergone fundamental transformations. In a dynamic global environment, specialization and collaboration have become the dominant paradigms. Flexibility and agility are vital for the survival and competitiveness of manufacturing firms, implying the ability to rapidly adapt to constantly changing markets and respond promptly to customer demands. However, the immobility of fixed facilities in traditional manufacturing enterprises limits these capabilities. With advances in modern manufacturing technologies, many manufacturers are adopting more contemporary strategies, focusing on their core competencies while outsourcing other activities, particularly those that require substantial investment but yield relatively low returns. This strategy benefits manufacturers in three main ways. First, it enhances flexibility and agility, as outsourcing can significantly shorten time-to-market. Second, reduced investment in machinery acquisition and plant construction lowers capital requirements and operational risks. Finally, firms can sustain higher competitiveness and profitability, since concentrating on core activities typically enables them to achieve higher returns with lower capital input.

Another major trend reshaping the manufacturing industry is the growing demand for customization. In cloud manufacturing systems, an order is equivalent to a task. In the past, consumers purchased finished products without participating in the design or production stages; this is no longer the case. Today, consumers increasingly demand products that meet their unique and personalized needs. The rising trend of customization is prevalent across nearly all industries, and manufacturing is no exception. Consequently, manufacturing enterprises must confront this challenge by transforming themselves into manufacturing service providers to further enhance their potential. Cloud manufacturing, which integrates design and manufacturing as services, offers a forward-looking solution to address these challenges.

Recent studies in cloud manufacturing systems (CMfgS) have increasingly focused on integrating operational, logistical, and customer-oriented decision-making processes. However, the existing literature remains fragmented, as most studies emphasize only limited subsets of scheduling, logistics, pricing, quality, or customer-related objectives. To better identify the research gap, the literature can be categorized into five main streams: logistics integration, scheduling optimization, pricing and economic decision-making, customer satisfaction and fairness considerations, and solution methodologies.

### 2.1. Logistics integration in cloud manufacturing

Logistics coordination is recognized as one of the key operational challenges in cloud manufacturing systems because manufacturing resources are geographically distributed and tasks often require inter-factory transportation. Several studies have investigated logistics-related scheduling decisions in CMfgS environments.

Zhou et al. [3] studied the logistics service selection (LSS) problem and proposed an optimization-based model to reduce delivery time compared with conventional routing strategies. Delaram and Valilai [4] investigated integrated logistics operations and service selection in cloud manufacturing environments by considering transportation-related decisions. Zhou et al. [5] focused on logistics scheduling with preselected manufacturers and aimed to minimize average delivery times between manufacturers and customers. Wu et al. [6] proposed the integrated cloud manufacturing order and logistics scheduling (ICSOLS) problem and developed a hybrid mathematical–metaheuristic solution approach to improve supply chain efficiency. Similarly, Zhao et al. [7] presented a collaborative optimization framework for simultaneously scheduling manufacturing and logistics services while minimizing order completion time and vehicle idle time.

Several additional studies also incorporated logistics into scheduling formulations. Li et al. [1], Liu et al. [8], and Zhang et al. [9] developed multi-objective models with explicit logistics considerations. Liu et al. [10] proposed a multi-task scheduling model integrating transportation, time, and cost objectives. Liu et al. [11] investigated integrated manufacturing and logistics scheduling using metaheuristic algorithms to jointly optimize makespan, tardiness, and cost. Zhang et al. [12] addressed integrated production and delivery scheduling across geographically dispersed factories using mixed-integer programming and heuristic approaches. Furthermore, Salmasnia and Kiapasha [13] extended the integrated subtask scheduling and logistics (ISSL) model by incorporating hybrid logistics, heterogeneous task arrivals, and parallel/sequential task structures.

Although these studies significantly improved logistics coordination in cloud manufacturing systems, most primarily focused on operational performance indicators such as delivery time, transportation cost, or makespan. Customer-oriented factors such as pricing policies, customer satisfaction, and fairness among customers were generally not integrated simultaneously within the optimization framework.

### 2.2. Scheduling optimization and resource allocation

Another major stream of research focuses on scheduling optimization and efficient resource allocation in cloud manufacturing systems. These studies mainly address machine assignment, task sequencing, service coordination, and dynamic scheduling.

Cao et al. [14] formulated a multi-objective scheduling problem considering time, cost, and service criteria together with logistics. Chen et al. [15] investigated customer coordination during service reservation and scheduling processes while considering completion times. Zhou et al. [2] proposed a dynamic multi-task scheduling model using queue-based service selection rules in cloud manufacturing systems. Yuan et al. [16] developed a dynamic service resource scheduling model considering time, cost, and reliability objectives.

Recent studies have increasingly adopted intelligent optimization techniques. Wang et al. [17] improved deep reinforcement learning (DRL)-based dynamic task scheduling by integrating data distribution into the loss function to enhance training efficiency. Hu et al. [18] proposed a DDDQN-based intelligent scheduling framework for stamping resources while considering task dependency and service-switching costs. Chen et al. [19] introduced a customer-oriented multi-task scheduling approach using a hyper-heuristic algorithm to maximize customer satisfaction and operational efficiency.

Other researchers emphasized operational balancing and performance optimization. Jafarnejad Ghomi et al. [20] developed a queuing-based load-balancing scheduling framework solved using a genetic algorithm. Ahn and Hur [21] proposed a model minimizing tardiness penalties and completion costs while maximizing quality and reliability. Wang et al. [22] addressed scheduling problems considering energy consumption, quality, reliability, workload balancing, machine idle time, and logistics-related objectives.

Despite the significant advances in scheduling optimization, most existing studies primarily concentrate on operational efficiency and resource allocation. Integrated consideration of pricing decisions, customer utility, and fairness among customers remains relatively limited in the current scheduling literature.

### 2.3. Pricing and economic decision-making

Pricing and economic optimization have also attracted growing attention in cloud manufacturing research because service providers seek to maximize profitability while maintaining competitiveness.

Han et al. [23] proposed a pricing and revenue optimization model for cloud manufacturing service providers using game-theoretic concepts. Meng and Xu [24] developed a pricing framework for determining pricing variables throughout the product life cycle. Vahedi-Nouri et al. [25] presented an integrated pricing and scheduling model aimed at improving customer satisfaction, manufacturer utility, and shareholder value simultaneously. In addition, a recent review by [26] comprehensively analyzed game theory-based decision-making models in production planning, scheduling, sustainable manufacturing, and cloud manufacturing systems. Their study highlighted the increasing importance of multi-agent interactions, pricing mechanisms, and collaborative decision-making in CMfgS environments. However, the review also emphasized that the simultaneous integration of customer satisfaction, fairness, logistics coordination, and pricing decisions within a unified optimization framework remains limited in the current literature.

Although these studies incorporated pricing-related decisions into cloud manufacturing environments, most did not jointly consider logistics coordination, fairness among customers, and product quality within a unified multi-objective optimization framework.

### 2.4. Customer satisfaction and fairness considerations

Customer-oriented decision-making has become increasingly important in modern cloud manufacturing systems due to growing demand for customized and high-quality products.

Chen et al. [19] focused on maximizing customer satisfaction through service availability management and time-related expectations. Vahedi-Nouri et al. [25] incorporated customer utility and shareholder value into cloud manufacturing scheduling decisions. Zhang et al. [9] proposed a utility-aware multi-task scheduling model using game theory and an extended NSGA-II algorithm. Liu et al. [27] also investigated customer-oriented project management models with logistics-related considerations. Furthermore, Zhang et al. [28] investigated resource scheduling optimization in cloud manufacturing by simultaneously considering both supply-side and demand-side service requirements. Their model incorporated workload balancing, customer satisfaction, cost, time, and vehicle-related constraints. Although the study provided a more balanced perspective between service providers and customers, fairness among customers, integrated pricing and product quality decisions were not explicitly considered.

Nevertheless, fairness among customers has received relatively limited attention in the existing literature. Most prior studies optimize average system-level performance measures without explicitly addressing equitable satisfaction distribution among customers. In addition, the simultaneous integration of customer satisfaction, fairness, pricing, logistics, quality, and tardiness/earliness penalties remains insufficiently explored.

A recent study by Salmasnia et al. [29] investigated the mutual benefits of cloud manufacturing systems and customers through the integration of scheduling, order acceptance, fairness, maintenance strategy, pricing, and earliness/tardiness considerations. Although their work shares several customer-oriented and economic decision-making aspects with the present study, product quality was not explicitly modeled as an independent decision-making criterion affecting customer satisfaction and system performance. In contrast, the proposed model in this study explicitly incorporates product quality together with pricing, logistics scheduling, customer satisfaction, and fairness within a unified multi-objective cloud manufacturing optimization framework. Furthermore, the proposed approach evaluates customer utility based not only on economic factors but also on quality-oriented performance measures in geographically distributed manufacturing environments.

### 2.5. Solution methodologies in cloud manufacturing

Various optimization and intelligent solution methodologies have been employed in the literature to solve cloud manufacturing problems. Exact optimization approaches such as mixed-integer programming were adopted by Akbaripour et al. [30], Delaram and Valilai [4], and Zhang et al. [12]. Metaheuristic approaches including genetic algorithms and hybrid heuristics were used by Jafarnejad Ghomi et al. [20], Salmasnia and Kiapasha [13], and Wu et al. [6]. More recently, intelligent learning-based methods such as DRL and DDDQN were introduced by Wang et al. [17] and Hu et al. [18], respectively.

Although these studies proposed efficient optimization approaches, the majority focused on operational objectives and did not simultaneously integrate economic, customer-oriented, and fairness-related dimensions within a comprehensive cloud manufacturing decision-making framework.

### 2.6. Research gap and contribution of the present study

A comprehensive review of studies on cloud manufacturing systems is summarized in Table 1. As can be seen in Table 1, most existing studies in cloud manufacturing systems have primarily focused on limited combinations of scheduling-related objectives, such as cost, tardiness, logistics, or resource allocation. Only a small number of studies simultaneously consider customer-oriented criteria together with operational and economic objectives. Moreover, the existing literature rarely integrates product quality, pricing decisions, customer satisfaction, fairness considerations, tardiness/earliness penalties, logistics integration, and heterogeneous manufacturing resources within a unified optimization framework.

**Table 1:** Literature review.

Research	Product Quality	Pricing	Fairness	Customer Satisfaction	Tardiness	Earliness	Type of Machines		Number of Tasks		Type of Subtasks	
							Identical	Different	Single	Multiple	Identical	Different
Wu et al. [6]					✓		✓			✓	✓	
Vahedi-Nouri et al. [25]		✓	✓	✓	✓			✓		✓		
Zhou et al. [5]							✓			✓	✓	
Chen et al. [15]					✓		✓			✓	✓	
Chen et al. [19]				✓				✓		✓		
Hu et al. [18]								✓		✓		✓
Zhang et al. [12]								✓				
Zhao et al. [7]					✓			✓		✓		✓
Yuan et al. [16]							✓			✓		✓
Wang et al. [22]					✓		✓			✓		
Wang et al. [17]								✓		✓		✓
Wan et al. [31]										✓		✓
Salmasnia and Kiapasha [13]								✓		✓		✓
Liu et al. [11]					✓		✓			✓	✓	
Jafarnejad Ghomi et al. [20]					✓		✓			✓	✓	
Jafarnejad Ghomi et al. [32]					✓		✓			✓	✓	
Akbaripour et al. [30]							✓		✓			
Liu et al. [27]										✓		
Li et al. (2015)							✓			✓		✓
Liu et al. [10]							✓			✓	✓	
Zhou et al. [2]							✓			✓		
Zhang et al. [9]							✓					
Ahn and Hur [21]	✓				✓		✓			✓		
Zhou et al. [3]							✓			✓		
Liu et al. [8]							✓			✓	✓	
Cao et al. [14]							✓		✓			
Delaram and Fatahi Valilai [4]							✓			✓		✓
Meng and Xu [24]		✓					✓					
Han et al. [23]		✓					✓					
Zhang et al. [28]				**✓**			**✓**			**✓**		**✓**
Salmasnia et al, [29]		**✓**	**✓**	**✓**	**✓**	**✓**		**✓**		**✓**		**✓**
**Proposed Model**	**✓**	**✓**	**✓**	**✓**	**✓**	**✓**		**✓**		**✓**		**✓**

The comparative analysis presented in Table 1 demonstrates that previous studies generally address only subsets of these dimensions. In contrast, the proposed model simultaneously incorporates all of these key aspects within a single multi-objective cloud manufacturing framework. Therefore, the main contribution of this study lies in providing a more comprehensive and balanced decision-making structure that jointly considers system-level profitability and customer-oriented performance measures.

Despite the considerable progress in cloud manufacturing research, several important gaps still remain. Existing studies have mainly focused on isolated or partially integrated aspects of cloud manufacturing decision-making, such as scheduling efficiency, logistics coordination, pricing strategies, quality improvement, or customer-oriented evaluation. Although some studies separately consider customer satisfaction, fairness, or pricing decisions, limited attention has been devoted to simultaneously modeling the interactions among pricing, product quality, customer satisfaction, fairness, logistics scheduling, and tardiness/earliness penalties within an integrated optimization framework.

Furthermore, most existing approaches primarily emphasize operational efficiency and system-oriented objectives, while the balance between cloud manufacturing profitability and customer-oriented performance measures has not been sufficiently investigated. Therefore, this study addresses this gap by proposing a unified multi-objective optimization framework that simultaneously integrates logistics operation scheduling, pricing decisions, product quality, customer satisfaction, fairness among customers, and tardiness/earliness considerations in cloud manufacturing systems.

Although customer segmentation, collaborative manufacturing behavior, and interoperability among heterogeneous cloud manufacturing participants are recognized as important research directions in CMfgS environments, these aspects are beyond the scope of the present study. The proposed model focuses specifically on the integrated optimization of scheduling, logistics, pricing, quality, customer satisfaction, and fairness within a centralized cloud manufacturing decision-making framework. Incorporating customer segmentation mechanisms and interoperability-related constraints into the optimization process represents a valuable direction for future research.

## 3. Problem definition

**Fig 1:**
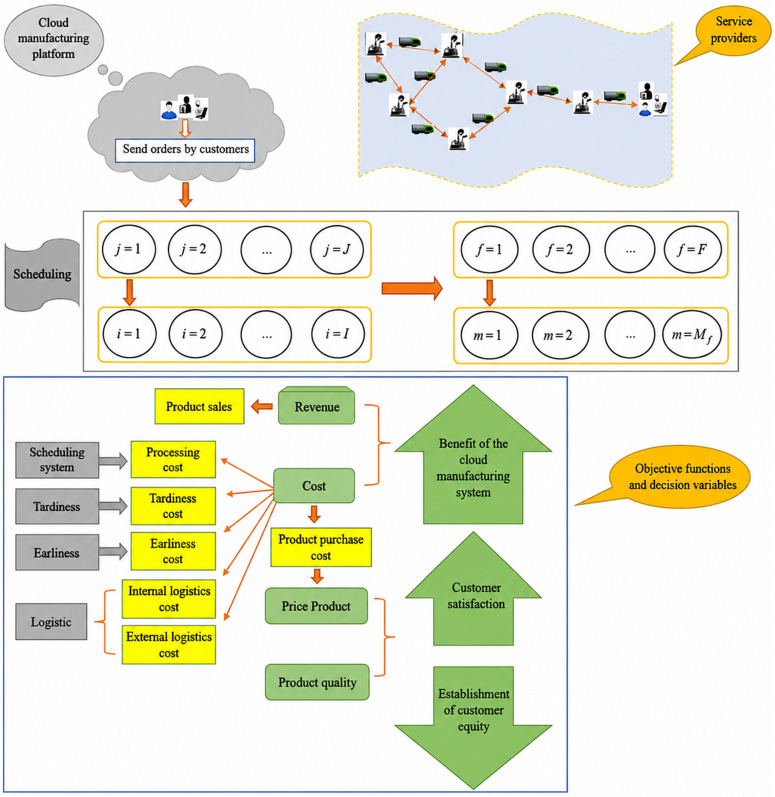
Framework of the problem process in the cloud manufacturing system.

Cloud manufacturing systems are driven by customer orders and can therefore be regarded as make-to-order production systems [25]. At the same time, this paradigm provides an appropriate platform for sharing manufacturing capabilities among industrial enterprises. In the problem considered in this study, on one side there is a set of customers (j∈J), and on the other side there is a set of factories located in different geographical regions. These factories possess the capabilities required to fulfill customer orders, which are shared through the cloud manufacturing system. As illustrated in Fig 1, orders are submitted by customers, who are generally interested in receiving their orders on time. Due to the limited capacity of system resources, processing one customer’s order may delay another order. Although cloud manufacturing systems can generate revenue by processing orders, they also face the risk of delivering orders after their due dates, which may result in penalty costs. Consequently, cloud manufacturing systems must select and process orders based on a careful analysis of available capacities, order due dates, and processing costs, while also accounting for tardiness penalties, earliness costs, and transportation costs, in order to maximize overall profit.

In a cloud manufacturing system, the processes from receiving a customer order to delivering the final product proceed as follows. First, the customer order is received; then, the cloud manufacturing platform analyzes the order (task) and decomposes it into subtasks. The subtasks are scheduled in such a way that the most appropriate machine is selected for each one. After assigning subtasks to machines, the actual manufacturing process begins, which includes execution, material handling, and logistics operations for transferring semi-finished products to subsequent factories. In this context, two important points should be noted: (1) when the next subtask is executed at the same production location, logistics services are not required; and (2) once the manufacturing process is completed, the final product must be delivered to the customer. Given the necessity of logistics services, the logistics provider that enables faster transportation between factories is selected. In other words, logistics time and cost, as well as logistics service selection, are explicitly considered in this study.

The second objective function represents customer satisfaction. Customer satisfaction is a function of the price that customers must pay for their orders as well as the quality of the products they receive. Customers prefer to obtain their required orders at the lowest possible price and the highest possible quality, while the cloud manufacturing system seeks to maximize its profit. Therefore, a model that simultaneously considers the interests of both parties can be highly effective. However, in most existing cloud manufacturing models, only the interests of the cloud manufacturing system are considered, often prioritizing system profitability over customer benefits.

The third objective function reflects the level of fairness in customer satisfaction, ensuring that satisfaction levels among customers do not differ significantly. For example, the model seeks to avoid situations in which one customer enjoys a very high level of satisfaction while another experiences a very low level. Hence, equality and fairness among customers are of particular importance for maintaining customer satisfaction and ensuring the survival and sustainable growth of cloud manufacturing systems. Accordingly, this paper defines a specific objective function to capture fairness among customers.

The assumptions used in the proposed models in this study are as follows:

- Each customer submits an order with specific characteristics to the cloud manufacturing system.
- Each customer has a due date for their order, and delivery after this time results in a delay or tardiness cost.
- All orders are available at the beginning of the planning horizon.
- Each factory is equipped with a set of machines $\left(m\in M_{f}\right)$ with different capabilities that can satisfy the technical requirements of subsets of orders; therefore, a given machine may not be capable of processing a specific order.
- p∈P denotes a specific position on a machine at which processing operations can be performed.
- There are no precedence or priority relationships among orders.
- Customer satisfaction can be evaluated based on influential factors such as price and product quality.

It should be emphasized that customer satisfaction in the proposed model is not derived from subjective survey-based evaluations or behavioral feedback data. Instead, satisfaction is formulated as an operational utility function based on measurable quantitative criteria, namely product price and delivered quality. This assumption is particularly appropriate in business-to-business cloud manufacturing environments, where service requesters evaluate manufacturing services primarily according to economic and technical performance indicators.

The symbols and definitions used for formulating the problem are provided in Table 2.

**Table 2:** Notations.

Indices:	
j	Task counter j=1,2,…,J
i	Subtask counter i=1,2,…,I
m	Machine counter m=1,2,…,M
l	Logistics service counter l=1,2,…,L
f	Factory counter f=1,2,…,F
p	Position counter for subtask sequences on a machine p=1,2,...,P
**Binary Decision Variables:**	
$x_{ijp}^{mf}$	1 if subtask i of task j is scheduled in position p on machine m of factory f; 0 otherwise.
$y_{j(i,i+\text{1})}^{l\left(f,f^{\prime }\right)}$	1 if logistics service l is used between factories f and f′ for transporting subtask i to i + 1 of task j; 0 otherwise.
**Continuous Decision Variables:**	
$S_{ij}$	Start time of subtask i of task j
$E_{ij}$	Completion time of subtask i of task j
$tard_{j}$	Tardiness duration of task j
$earl_{j}$	Earliness duration of task j
${Pr}_{j}$	Price of task j
$d_{j}$	Customer satisfaction (utility) of task j
$d_{max}$	Maximum utility value among all customer tasks
$\Delta d_{j}$	dmax−dj
$Q_{j}$	Product quality of task j
$ETC_{j}$	Total average cost of customer j
$TC_{CMfg}$	Total cost incurred by the cloud manufacturing system
$TR_{CMfg}$	Total revenue of the cloud manufacturing system
$ETP_{CMfg}$	Net profit of the cloud manufacturing system
**Cost and Time Parameters for Subtasks:**	
$PT_{ij}^{mf}$	Processing time required for subtask i of task j on machine m of factory f (in time units)
$PC_{ij}^{mf}$	Processing cost for subtask i of task j on machine m of factory f (in monetary units)
$MAC_{j}$	Maximum acceptable cost that customer j is willing to incur in the cloud manufacturing system
**Logistics Parameters:**	
$d_{f,f^{\prime }}$	Distance of the logistics service between two factories f and f′
$b_{l}$	Logistics cost per logistics service
$k_{l}$	Logistics time per logistics service
$p_{end}$	Indicates the necessity of using a logistics service from the factory where the last subtask was completed to the delivery location (customer), i.e., where transportation ends
$LC_{f,f^{\prime }}^{l}$	Logistics service cost l between two factories f and f′
$LC_{f,p_{end}}^{l}$	Logistics service cost l from factory f to customer location$p_{end}$
$LT_{f,f^{\prime }}^{l}$	Time required for logistics service l between two factories f and f′
$LT_{f,p_{end}}^{l}$	Time required for logistics service l from factory f to customer location $p_{end}$
**Due Date Parameters:**	
$DU_{j}$	Due date of task j
$\omega_{j}$	Penalty per unit of tardiness for task j
$\pi_{j}$	Penalty per unit of earliness for task j
$M_{\text{1}}-M_{\text{3}}$	A large positive number
**Utility Parameter:**	
α	Minimum utility obtained by a customer
**Quality Parameters:**	
$q_{i}^{f}$	Quality of the final subtask i from factory f
$Q_{j}^{min}$	Minimum acceptable quality level specified by the customer
**Binary Parameters:**	
$\Gamma_{ij}^{mf}$	1 if machine m from factory f is capable of processing subtask i of task j, otherwise 0

## 4. Mathematical modeling

Given that customers prefer lower total costs for their orders while the cloud manufacturing system seeks higher profit, the first objective function is defined as the profit of the cloud manufacturing system.

### 4.1. Total cost of the cloud manufacturing system

The total cost of the cloud manufacturing system is obtained from the sum of $PC_{ij}^{mf}$, the processing cost, $LC_{f,f^{\prime }}^{l}$, the logistics service cost between two factories f and $f^{\prime }$, $LC_{f,p_{end}}^{l}$, the logistics service cost from factory f to the customer location $p_{end}$, and the total tardiness ($tard_{j}$) and earliness ($earl_{j}$) durations, as defined in Equation (1).This total cost will then be used to calculate the system profit by subtracting it from the revenue generated by processing orders.


$$\begin{array}{cc} TC_{CMfg}= & \sum_{i\in I}\sum_{j\in J}\sum_{p\in P}\sum_{m\in M_{f}}\sum_{f\in F}x_{ijp}^{mf}PC_{ij}^{mf}+\sum_{i\in I}\sum_{j\in J}\sum_{l\in L}\sum_{f,f^{\prime }\in F}y_{j(i,i+\text{1})}^{l\left(f,f^{\prime }\right)}LC_{f,f^{\prime }}^{l}\\ & +\sum_{i\in I}\sum_{j\in J}\sum_{l\in L}\sum_{\begin{array}{cc} & f,p_{end}\in F\\ & f\neq p_{end} \end{array}}y_{j(i,end)}^{l\left(f,p_{end}\right)}LC_{f,p_{end}}^{l}+\sum_{j\in J}\left(tard_{j}\omega_{j}+earl_{j}\pi_{j}\right) \end{array}$$ (1)

In this problem, $b_{l}$ represents the logistics service cost, and $k_{l}$ denotes the logistics service time, which is calculated based on the distance between two factories using the following relationships.


$$\begin{array}{c} LC_{f,f^{\prime }}^{l}=b_{l}\times d_{f,f^{\prime }} \end{array}$$ (2)

$$\begin{array}{c} \;LT_{f,f^{\prime }}^{l}=k_{l}\times d_{f,f^{\prime }} \end{array}$$ (3)

### 4.2. Total revenue of the cloud manufacturing system

The selling price of each unit of product is denoted by ${Pr}_{j}$, where ${Pr}_{j}$ represents the selling price for order j. Accordingly, the average revenue of the cloud manufacturing system is calculated using the following relationship.


$$\begin{array}{c} TR_{CMfg}=\sum_{j\in J}{Pr}_{j} \end{array}$$ (4)

### 4.3. Final profit of the cloud manufacturing system

By subtracting the total cost of the cloud manufacturing system $TC_{CMfg}$ from the total revenue $TR_{CMfg}$, the net profit of the cloud manufacturing system $ETP_{CMfg}$ is calculated using the following relationship.


$$\begin{array}{c} ETP_{CMfg}=TR_{CMfg}-TC_{CMfg} \end{array}$$ (5)

## 4.4. Customer cost analysis

The customer’s cost includes the purchase cost of the product. Therefore, the total cost for customer j over the product life cycle $ETC_{j}$ is calculated as follows.


$$\begin{array}{c} ETC_{j}={Pr}_{j}\;\forall j\in J \end{array}$$ (6)

### 4.5. Customer satisfaction

Customer satisfaction is a function of the cost incurred by the customer and the quality of the product. Therefore, the overall quality of the product, $Q_{ij}$, is equal to $q_{i}^{f}$, which represents the quality of the subtask i completed at factory f, and is calculated using the following relationship.


$$\begin{array}{c} Q_{j}=\sum_{i\in I}\sum_{p\in P}\sum_{m\in M_{f}}\sum_{f\in F}q_{I}^{f}x_{ijp}^{mf}\;\forall j\in J \end{array}$$ (7)

Since the units of price and quality are different, it is necessary to normalize both factors to make them dimensionless. Therefore, we have:


$$\begin{array}{c} Q_{j}^{n}=\frac{Q_{j}-Q_{j}^{min}}{Q_{j}^{max}-Q_{j}^{min}}\;\forall j\in J \end{array}$$ (8)

$Q_{j}^{min}$is obtained when each subtask i of order j is processed by the factory with the lowest quality, whereas $Q_{j}^{max}$ corresponds to the scenario in which all subtasks are executed at factories with the highest quality.

## 4.6. Objective functions and constraints

Based on the explanations above, the objective functions and constraints of the proposed model can be formulated as follows:


$$\begin{array}{c} maxZ_{\text{1}}=ETP_{CMfg} \end{array}$$ (9)

$$\begin{array}{c} maxZ_{\text{2}}=\sum_{j\in J}d_{j} \end{array}$$ (10)

$$\begin{array}{c} minZ_{\text{3}}=\sum_{j\in J}\Delta d_{j} \end{array}$$ (11)

$$\begin{array}{c} s.t. \end{array}$$

$$\begin{array}{c} \sum_{p\in P}\sum_{m\in M_{f}}\sum_{f\in F}x_{ijp}^{mf}=\text{1}\;\forall i\in I,j\in J \end{array}$$ (12)

$$\begin{array}{c} \sum_{i\in I}\sum_{j\in J}x_{ijp}^{mf}\leq \text{1}\;\forall p\in P,\forall m\in M_{f},f\in F \end{array}$$ (13)

$$\begin{array}{c} \sum_{i\in I}\sum_{j\in J}x_{ijp}^{mf}\leq \sum_{i\in I}\sum_{j\in J}x_{ij(P-\text{1})}^{mf}\;\forall p\in P,p\neq \text{1},\forall m\in M_{f},f\in F \end{array}$$ (14)

$$\begin{array}{c} \sum_{p\in P}x_{ijp}^{mf}\leq \Gamma_{ij}^{mf}\;\forall i\in I,j\in J,\forall m\in M_{f},f\in F \end{array}$$ (15)

$$\begin{array}{c} S_{ij}\geq E_{i^{\prime }j^{\prime }}-M_{\text{1}}\times \left(\text{2}-x_{i^{\prime }j^{\prime }(p-\text{1})}^{mf}-x_{ijp}^{mf}\right)\;\forall i,i^{\prime }\in I,j,j^{\prime }\in J,if\left(j=j^{\prime }then.i^{\prime }<i\right) \end{array}$$ (16)

$$\begin{array}{c} \forall p\in P,p\neq \text{1},\forall m\in M_{f},f\in F \end{array}$$

$$\begin{array}{c} S_{ij}\geq E_{(i-\text{1})j}+\sum_{l\in L}\sum_{\begin{array}{cc} & f,f^{\prime }\in F\\ & f\neq f^{\prime } \end{array}}LT_{f,f^{\prime }}^{l}\times y_{j(i,i+\text{1})}^{l\left(f,f^{\prime }\right)}\;\forall i\in I,j\in J \end{array}$$ (17)

$$\begin{array}{c} \;E_{ij}\geq S_{ij}+\sum_{p\in P}\sum_{m\in M_{f}}\sum_{f\in F}x_{ijp}^{mf}\times PT_{ij}^{mf}\;\forall i\in I,j\in J \end{array}$$ (18)

$$\begin{array}{c} x_{ijp}^{mf}+x_{(i+\text{1})j^{\prime }p^{\prime }}^{m^{\prime }f^{\prime }}-\text{1}\leq \sum_{l\in L}y_{j(i,i+\text{1})}^{l\left(f,f^{\prime }\right)}\;\forall i\in I,j\in J,\forall p,p^{\prime }\in P \end{array}$$

$$\begin{array}{c} \forall m,m^{\prime }\in M_{f},\forall f,f^{\prime }\in F,f\notin f^{\prime } \end{array}$$ (19)

$$\begin{array}{c} x_{Ijp}^{mf}\leq \sum_{l\in L}y_{j(I,end)}^{l\left(f,p_{end}\right)}\;\forall I\in I,j\in J,\forall p\in P,\forall m\in M_{f}, \end{array}$$ (20)

$$\begin{array}{c} \forall f\in F,f\text{≠}p_{end} \end{array}$$

$$\begin{array}{c} \;tard_{j}\geq \left(\sum_{I\in I}E_{Ij}+\sum_{i\in I}\sum_{l\in L}\sum_{\begin{array}{cc} & f\in F\\ & f\text{≠}p_{end} \end{array}}LT_{f,p_{end}}^{l}\times y_{j(I,end)}^{l\left(f,p_{end}\right)}\right)-DU_{j} \end{array}$$ (21)

$$\begin{array}{c} \forall j\in J \end{array}$$

$$\begin{array}{c} earl_{j}\geq DU_{j}-\left(\sum_{I\in I}E_{Ij}+\sum_{i\in I}\sum_{l\in L}\sum_{\begin{array}{cc} & f\in F\\ & f\text{≠}p_{end} \end{array}}LT_{f,p_{end}}^{l}\times y_{j(I,end)}^{l\left(f,p_{end}\right)}\right) \end{array}$$ (22)

$$\begin{array}{c} \forall j\in J \end{array}$$

$$\begin{array}{c} {Pr}_{j}\geq \sum_{i\in I}\sum_{p\in P}\sum_{m\in M_{f}}\sum_{f\in F}x_{ijp}^{mf}PC_{ij}^{mf}+\sum_{i\in I}\sum_{l\in L}\sum_{f,f^{\prime }\in F}y_{j(i,i+\text{1})}^{l\left(f,f^{\prime }\right)}LC_{f,f^{\prime }}^{l} \end{array}$$

$$\begin{array}{c} +\sum_{i\in I}\sum_{l\in L}\sum_{\begin{array}{cc} & f,p_{end}\in F\\ & f\text{≠}p_{end} \end{array}}y_{j(i,end)}^{l\left(f,p_{end}\right)}LC_{f,p_{end}}^{l}+tard_{j}\omega_{j}+earl_{j}\pi_{j} \end{array}$$ (23)

$$\begin{array}{c} \forall j\in J \end{array}$$

$$\begin{array}{c} \;{Pr}_{j}\leq \left(\sum_{i\in I}\sum_{p\in P}\sum_{m\in M_{f}}\sum_{f\in F}x_{ijp}^{mf}\right)\times M_{\text{2}}\;\forall j\in J \end{array}$$ (24)

$$\begin{array}{c} ETC_{j}={Pr}_{j}\;\forall j\in J \end{array}$$ (25)

$$\begin{array}{c} ETC_{j}\leq MAC_{j}\;\forall j\in J \end{array}$$ (26)

$$\begin{array}{c} \;TR_{CMfg}=\sum_{j\in J}{Pr}_{j} \end{array}$$ (27)

$$\begin{array}{c} TC_{CMfg}=\sum_{i\in I}\sum_{j\in J}\sum_{p\in P}\sum_{m\in M_{f}}\sum_{f\in F}x_{ijp}^{mf}PC_{ij}^{mf}+\sum_{i\in I}\sum_{j\in J}\sum_{l\in L}\sum_{f,f^{\prime }\in F}y_{j(i,i+\text{1})}^{l\left(f,f^{\prime }\right)}LC_{f,f^{\prime }}^{l} \end{array}$$ (28)

$$\begin{array}{c} +\sum_{i\in I}\sum_{j\in J}\sum_{l\in L}\sum_{\begin{array}{cc} & f,p_{end}\in F\\ & f\text{≠}p_{end} \end{array}}y_{j(i,end)}^{l\left(f,p_{end}\right)}LC_{f,p_{end}}^{l}+\sum_{j\in J}\left(tard_{j}\omega_{j}+earl_{j}\pi_{j}\right) \end{array}$$

$$\begin{array}{c} ETP_{CMfg}=TR_{CMfg}-TC_{CMfg} \end{array}$$ (29)

$$\begin{array}{c} \;Q_{j}=\sum_{i\in I}\sum_{p\in P}\sum_{m\in M_{f}}\sum_{f\in F}q_{i}^{f}x_{ijp}^{mf}\;\forall j\in J \end{array}$$ (30)

$$\begin{array}{c} Q_{j}\leq \sum_{i\in I}\sum_{p\in P}\sum_{m\in M_{f}}\sum_{f\in F}x_{ijp}^{mf}\times M_{\text{3}}\;\forall j\in J \end{array}$$ (31)

$$\begin{array}{c} Q_{j}\geq Q_{j}^{min}\;\forall j\in J \end{array}$$ (32)

$$\begin{array}{c} d_{j}\leq \alpha +(\text{1}-\alpha )\times \left[\frac{\frac{MAC_{j}-{Pr}_{j}}{MAC_{j}-C_{j}^{*}}+\frac{Q_{j}-Q_{j}^{min}}{Q_{j}^{max}-Q_{j}^{min}}}{\text{2}}\right]\;\forall j\in J \end{array}$$ (33)

$$\begin{array}{c} C_{j}^{*}=min\left\{\sum_{i\in I}\sum_{p\in P}\sum_{m\in M_{f}}\sum_{f\in F}x_{ijp}^{mf}PC_{ij}^{mf}+\sum_{i\in I}\sum_{l\in L}\sum_{f,f^{\prime }\in F}y_{j(i,i+\text{1})}^{l\left(f,f^{\prime }\right)}LC_{f,f^{\prime }}^{l}+\sum_{i\in I}\sum_{l\in L}\sum_{\begin{array}{cc} & f,p_{end}\in F\\ & f\neq p_{end} \end{array}}y_{j(i,end)}^{l\left(f,p_{end}\right)}LC_{f,p_{end}}^{l}\right\}\;\forall j\in J \end{array}$$ (34)

$$\begin{array}{c} d_{max}\geq d_{j}\;\forall j\in J \end{array}$$ (35)

$$\begin{array}{c} \Delta d_{j}=d_{max}{-d}_{j}\;\forall j\in J \end{array}$$ (36)

$$\begin{array}{c} x_{ijp}^{mf}\in \left\{\text{0},\text{1}\right\}\;\forall i\in I,j\in J,p\in P \end{array}$$ (37)

$$\begin{array}{c} \forall m\in M_{f},f\in F \end{array}$$

$$\begin{array}{c} y_{j(i,i+\text{1})}^{l\left(f,f^{\prime }\right)}\in \left\{\text{0},\text{1}\right\}\;\forall i\in I,\;j\in J,\;\forall l\in L \end{array}$$ (38)

$$\begin{array}{c} \forall f,f^{\prime }\in F,f\text{≠}f^{\prime } \end{array}$$

$$\begin{array}{c} \;S_{ij},E_{ij}\geq \text{0}\;\forall i\in I,j\in J \end{array}$$ (39)

$$\begin{array}{c} \;tard_{j},earl_{j},d_{j},\Delta d_{j},Q_{j},{Pr}_{j},ETC_{j}\geq \text{0}\;\forall j\in J \end{array}$$ (40)

$$\begin{array}{c} d_{max},Q_{j}^{min},Q_{j}^{max}\geq \text{0} \end{array}$$ (41)

$$\begin{array}{c} \;TR_{CMfg},TC_{CMfg}\geq \text{0} \end{array}$$ (42)

Objective functions (9) and (10) represent, respectively, the maximization of the cloud manufacturing system’s profit and the total customer utility. Objective function (11) seeks fairness among customers, minimizing the total deviations between the highest customer utility and those of all other customers. Constraint (12) ensures that each subtask is performed exactly once on a sequence of a machine in a single factory, while constraint (13) prevents more than one subtask from the same order from being scheduled on the same machine sequence. Constraint (14) specifies that if the previous sequence of a machine is not assigned to a subtask, the next sequence cannot be scheduled. Constraint (15) limits each subtask to machines capable of meeting its technical requirements. Constraint (16) sets the start time of a subtask based on the completion of the previous subtask on the same machine, and constraint (17) ensures that a subtask cannot start until the previous subtask of the same order and its logistics are completed. Constraints (18) and (19) calculate the completion times of all subtasks for each order in a sequential structure, and constraint (20) requires the use of a logistics service from the factory where the last subtask was executed to the delivery point. Constraints (21) and (22) determine the tardiness and earliness of each order, while constraints (23) and (24) represent the total customer cost, including tardiness/earliness, internal and external logistics, and processing costs. Constraint (25) calculates the total cost of a customer’s order based on the purchase price, and constraint (26) ensures it does not exceed the maximum acceptable cost for that customer. Constraints (27) and (28) calculate the total revenue and total cost of the cloud manufacturing system, respectively, and constraint (29) derives the net profit by subtracting total cost from total revenue. Constraints (30) and (31) compute the overall product quality, which varies depending on the factory, while constraint (32) ensures the quality meets the customer’s minimum acceptable level. Constraints (33) and (34) model customer satisfaction as a function of normalized price and quality, and constraint (35) ensures that the maximum utility among all customers is higher than the utility of a given order. Constraint (36) calculates $\Delta d_{j}$ to measure fairness among customers, and constraints (37)–(42) define the decision variable types.

The proposed model thus includes three objectives: ETP (cloud manufacturing system profit), $d_{j}$ (customer satisfaction), and $\Delta d_{j}$ (fairness among customers). The LP-metric approach is used to combine these multiple objectives into a single composite objective function, as represented in equation (43).


$$\begin{array}{c} \;Total=w_{\text{1}}\left(\frac{ETP^{*}-ETP}{ETP^{*}}\right)+w_{\text{2}}\left(\frac{{d_{j}}^{*}-d_{j}}{{d_{j}}^{*}}\right)-w_{\text{3}}\left(\frac{\Delta {d_{j}}^{*}-\Delta d_{j}}{\Delta {d_{j}}^{*}}\right) \end{array}$$ (43)

## 5. Computational results

### 5.1. Illustrative examples

In this section, in order to illustrate the preformance of the proposed model, first a numerical example is presented and discussed in detail. The set values used in this example are summarized in Table 3. The task structure in this example consists of 3 orders, each of which contains 6 subtasks. The input parameter values for the problem are presented in Table 4.

**Table 3:** Set Values in the proposed model.

Indeces	Values
i	6
j	3
f	3
m	5
l	4
p	5

**Table 4:** Parameter values in the proposed model.

Parameters	Values
$PC_{ij}^{mf}$	(5,10)
γ	(0,1)
$MAC_{j}$	(220,250)
$\omega_{j}$	(1,2)
$\varphi_{j}$	(1,3)
$DU_{j}$	(10,20)
α	(0,1)
$PT_{ij}^{mf}$	(10,20)
$b_{l}$	(10,20)
$k_{l}$	(10,30)
$d_{f,f^{\prime }}$	(1,2)
$q_{i}^{f}$	(200,300)

**Fig 2:**
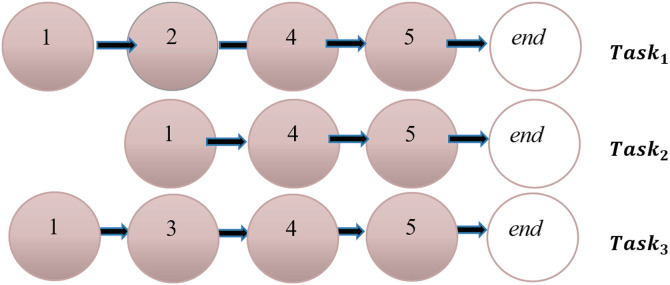
Task structure.

**Fig 3:**
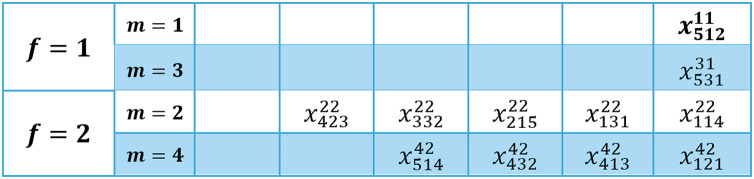
Assignment of subtasks to factory machines.

The task structure is presented in Table 5, which indicates the assignment of each subtask to its corresponding order. Table 6 shows the factory structure, specifying which machines are available in each factory. Fig 2 illustrates the sequential task structure. The overall objective function value for this example is 0.345. After solving the problem, the allocation of subtasks to each machine in each factory is depicted in Fig 3.

**Table 5:** Task structure.

Machine	$f_{1}$	$f_{2}$	$L_{end}$
1	✓		
2		✓	
3	✓		
4		✓	
5	✓		

**Table 6:** Factory structure.

Subtask	$Task_{1}$	$Task_{1}$	$Task_{1}$
1	✓	✓	✓
2			✓
3	✓		
4	✓	✓	✓
5	✓	✓	✓
end	✓	✓	✓

Tables 7 and 8 present the start and finish times of each subtask, while Table 9 provides details on logistics, including the selection of logistics services between factories and the delivery of the final subtask of each order to the delivery point. Early and tardiness values for the orders are shown in Table 10, indicating that all three orders experience some level of tardiness. The objective function values for the cloud manufacturing system’s profit, customer satisfaction, fairness among customers, and the overall objective function are summarized in Table 11.

**Table 10:** Task Tardiness and Earliness.

	Task 1	Task 2	Task 3
Tardiness	121.363	103.950	91.330
Earliness	0	0	0

**Table 11:** Objective Functions.

Objective Functions	Cloud Manufacturing System Profit	Customer Satisfaction	Fairness Among Customers	Overall Objective Function
Value	1222.191	1.960	0.865	0.345

**Table 7:** Subtask start timestimes.

Subtask	$Task_{1}$	$Task_{2}$	$Task_{3}$
1	48.062		
2	58.420		
3			15.079
4	73.978	29.211	29.211
5	89.976	68.768	58.758

**Table 8:** Subtask completion times.

Subtask	$Task_{1}$	$Task_{2}$	$Task_{3}$
1	58.420	16.345	15.079
2	73.987		
3			29.211
4	89.976	48.062	40.138
5	101.713	86.057	70.600

**Table 9:** Logistics Selection.

Inter-Factory Logistics	Logistics for Delivering the Final Subtask of Each Task to the Customer Delivery Point
$y_{j\left(i,i+1\right)}^{l\left(f,f^{\prime }\right)}$	$y_{j(I,end)}^{l\left(f,p_{end}\right)}$
$y_{245}^{121}$	$y_{\text{1}\text{5}\text{6}}^{\text{1}\text{2}\text{3}}$
$y_{345}^{121}$	$y_{\text{2}\text{5}\text{6}}^{\text{1}\text{1}\text{3}}$
	$y_{\text{3}\text{5}\text{6}}^{\text{1}\text{1}\text{3}}$

To further evaluate the practical applicability of the proposed model, an additional computational scalability analysis was conducted. The objective of this analysis is to investigate the behavior of the model under different problem sizes and to assess the computational requirements of the exact CPLEX solution approach when the dimensions of the cloud manufacturing system increase.

For this purpose, a set of representative instances was generated and classified into three categories: small-scale, medium-scale, and large-scale problems. The classification was based on the number of tasks, subtasks, factories, machines, and logistics services involved in the cloud manufacturing environment.

Table 12 summarizes the characteristics of all test instances together with the corresponding objective function values. As the problem size increases, the cloud manufacturing system profit and customer satisfaction generally increase due to the larger number of orders and available resources. However, the optimization problem also becomes significantly more complex because of the growing number of assignment, scheduling, logistics, pricing, and fairness decisions that must be determined simultaneously.

**Table 12:** Summary of example solutions using GAMS.

Problem Size	Example	Number of Tasks	Number of Subtasks	Number of Factories	Number of Machines	Number of Logistics Services	Cloud Manufacturing System Profit	Customer Satisfaction	Fairness Among Customers	Overall Objective Function
Small	S1	2	4	3	5	4	932.409	1.564	0.541	0.288
	S2	3	6	3	5	4	1222.191	1.960	0.865	0.345
	S3	5	7	4	5	4	2220.221	4.509	1.201	0.749
Medium	M1	6	12	5	8	5	2786.514	5.133	1.326	0.811
	M2	7	15	6	10	5	3348.772	6.015	1.447	0.925
	M3	8	18	7	12	6	3915.694	6.784	1.582	1.034
Large	L1	10	30	9	15	6	5210.348	8.613	1.844	1.216
	L2	12	36	10	18	7	5986.115	9.472	1.993	1.354
	L3	15	45	12	20	8	7148.387	11.058	2.231	1.518

To provide a detailed evaluation of computational performance, Table 13 reports the run time, number of decision variables, and number of constraints for each test instance. The results indicate that the proposed mixed-integer programming model can efficiently solve small- and medium-scale instances to optimality within acceptable computational times. For the small-scale instances, all solutions were obtained in less than one minute. Medium-scale instances required longer computational times because of the substantial increase in binary scheduling and logistics variables.

**Table 13:** Computational performance and scalability analysis.

Problem Size	Example	Run Time (sec)	Number of Variables	Number of Constraints
Small	S1	4.7	1,486	2,214
	S2	18.3	3,975	5,862
	S3	54.6	8,921	12,746
Medium	M1	182.4	24,618	34,581
	M2	614.8	48,305	67,924
	M3	1,874.2	87,446	122,307
Large	L1	>3600	312,588	438,942
	L2	>3600	596,731	837,516
	L3	>3600	1,184,965	1,662,843

For large-scale instances, the model size grows rapidly as the number of tasks, subtasks, factories, and logistics alternatives increases. Consequently, the exact CPLEX approach was unable to obtain proven optimal solutions within the predefined time limit of 3,600 seconds for the largest instances. This behavior is expected for integrated scheduling and logistics optimization models that contain a large number of binary variables and sequencing constraints. The results therefore demonstrate that the proposed exact optimization framework is appropriate for small- and medium-scale cloud manufacturing systems, while large-scale applications may require metaheuristic algorithms to obtain high-quality solutions within reasonable computational times.

### 5.2. Genetic algorithm evaluation for large-scale instances

The computational scalability analysis presented in the previous subsection demonstrated that the exact CPLEX solver can efficiently solve small- and medium-scale instances. However, as the problem size increases, the number of binary assignment, sequencing, and logistics decision variables grows significantly, resulting in a substantial increase in computational effort. For the largest test instances, CPLEX was unable to obtain proven optimal solutions within the predefined time limit of 3,600 seconds. Therefore, a Genetic Algorithm (GA) was developed to evaluate the applicability of the proposed model for large-scale cloud manufacturing environments.

The chromosome representation was designed to encode subtask assignments, machine selections, factory allocations, and logistics decisions simultaneously. Tournament selection was used for parent selection, while one-point crossover and mutation operators were employed to generate new solutions. Based on preliminary experiments, the GA parameters were set as follows: population size = 100, crossover probability = 0.80, mutation probability = 0.10, elitism rate = 5%, and tournament size = 3. The stopping criterion was defined as either reaching 500 generations or observing no improvement in the best solution for 50 consecutive generations.

To evaluate the performance of the proposed GA, the same nine test instances used in the scalability analysis were solved. Table 14 compares the objective function values and computational times obtained by the exact CPLEX solver and the proposed GA.

**Table 14:** Comparison between CPLEX and Genetic Algorithm.

Problem Size	Example	CPLEX Objective	GA Objective	Gap (%)	CPLEX Time (s)	GA Time (s)
Small	S1	0.288	0.288	0.00	12	21
	S2	0.345	0.345	0.00	38	54
	S3	0.749	0.749	0.00	57	81
Medium	M1	0.824	0.815	1.09	241	137
	M2	0.913	0.897	1.75	487	211
	M3	1.025	1.001	2.34	1185	462
Large	L1	N/A*	1.184	–	>3600	734
	L2	N/A*	1.276	–	>3600	1086
	L3	N/A*	1.391	–	>3600	1648

*CPLEX could not obtain a proven optimal solution within the 3,600-second time limit.

The results indicate that the exact CPLEX solver remains the most effective solution method for small-scale instances. In Examples S1, S2, and S3, both methods obtained identical objective function values, confirming the correctness of the GA implementation. Nevertheless, the computational times of the GA were slightly higher because the population-based search process requires multiple iterations even for relatively simple instances.

For medium-scale instances, the computational advantage of the GA becomes evident. While the exact solver still obtains optimal solutions, computational times increase considerably as the problem size grows. In contrast, the GA reduces solution times by approximately 40–60% while maintaining very small optimality gaps. The maximum gap observed among the medium-scale instances is only 2.34%, indicating that high-quality solutions can be obtained with substantially lower computational effort.

The benefits of the GA become even more significant for large-scale instances. As the number of tasks, subtasks, factories, and logistics alternatives increases, the exact mixed-integer programming model becomes extremely difficult to solve. Consequently, CPLEX was unable to prove optimality within the available time limit for Examples L1–L3. In contrast, the proposed GA successfully generated feasible high-quality solutions for all large-scale instances within reasonable computational times ranging from 734 to 1,648 seconds.

These results demonstrate that the exact optimization model is well suited for small- and medium-scale cloud manufacturing systems, where optimal solutions can be obtained within acceptable computational times. However, for realistic large-scale cloud manufacturing environments involving numerous manufacturing resources and logistics alternatives, metaheuristic approaches such as the proposed Genetic Algorithm provide a practical and computationally efficient alternative. Therefore, the GA can be considered a promising solution approach for large-scale applications of the proposed model and may serve as a foundation for future hybrid optimization frameworks combining exact and metaheuristic methods.

### 5.3. Comparative study

This section evaluates the effectiveness of the proposed model by examining the impact of excluding earliness/tardiness penalties, logistics decisions, and customer satisfaction considerations. The objective is to quantify the contribution of each modeling component to the overall performance of the cloud manufacturing system.

Table 15 presents a comparison between the proposed model and a model without considering earliness and tardiness penalties. The proposed model achieves a cloud manufacturing system profit of 1222.191, customer satisfaction of 1.960, fairness value of 0.865, and an overall objective function value of 0.345. When earliness and tardiness penalties are removed, profit increases slightly to 1236.514 and customer satisfaction increases marginally to 1.989, while fairness improves to 0.862. However, the overall objective function decreases to 0.341. Furthermore, evaluating the proposed model using the decision variables obtained from the model without earliness and tardiness results in an overall objective value of 0.469. Compared with the proposed solution, this alternative solution generates a 6.04% reduction in profit, a 6.52% reduction in customer satisfaction, and a 3.82% deterioration in fairness. In addition, total tardiness cost increases from 316.643 to 519.366. These results indicate that ignoring earliness and tardiness changes the scheduling behavior and may lead to solutions that are less balanced with respect to the multiple objectives considered in the model. Therefore, due-date related penalties play an important role in achieving an effective trade-off between profitability, customer satisfaction, and fairness.

**Table 15:** Comparison results between the proposed model and the model without considering earliness and tardiness penalties.

Model	Cloud Manufacturing System Profit	Customer Satisfaction	Fairness Among Customers	Overall Objective Function	Total Product Delivery Logistics Cost	Total Inter-Factory Logistics Cost	Product Quality	Total Tardiness Cost	Total Earliness Cost
Proposed Model	1222.191	1.96	0.865	0.345	12.231	74.588	2484.292	316.643	0
Without Considering Earliness and Tardiness	1236.514	1.989	0.862	0.341	12.243	74.653	2497.361	–	–
Expected Result Without Earliness and Tardiness	1152.541	1.84	0.898	0.469	13.654	75.021	2497.361	519.366	0
Improvement Percentage	6.04%	6.52%	3.82%	35.94%	11.63%	0.58%	−0.52%	64.02%	0.00%

Table 16 compares the proposed model with a model that ignores logistics decisions. Under the proposed framework, the overall objective function reaches 0.345, whereas removing logistics reduces it to 0.321. Although the model without logistics reports a slightly higher profit of 1240.265 and customer satisfaction of 1.998, the resulting schedule cannot realistically account for transportation activities between factories and delivery locations. When the decision variables obtained from the no-logistics model are evaluated within the proposed framework, total inter-factory logistics cost rises to 35.587 and delivery logistics cost increases to 107.943. These values correspond to increases of 190.96% and 44.72%, respectively, compared with the proposed model. Moreover, fairness deteriorates from 0.865 to 0.903. The results demonstrate that explicitly incorporating logistics enables the model to coordinate production and transportation decisions simultaneously, leading to more practical schedules and lower overall operational costs. Consequently, logistics should be considered an essential component of cloud manufacturing systems involving geographically distributed resources.

**Table 16:** Comparison results between the proposed model and the model without considering logistics.

Model	Cloud Manufacturing System Profit	Customer Satisfaction	Fairness Among Customers	Overall Objective Function	Total Product Delivery Logistics Cost	Total Inter-Factory Logistics Cost	Product Quality	Total Tardiness Cost	Total Earliness Cost
Proposed Model	1222.191	1.96	0.865	0.345	12.231	74.588	2484.292	316.643	0
Without Considering Logistics	1240.265	1.998	0.858	0.321	–	–	2664.348	301.254	–
Expected Result Without Logistics	1148.654	1.8	0.903	0.371	35.587	107.943	2664.348	305.541	0
Improvement Percentage	6.40%	8.89%	4.39%	7.54%	190.96%	44.72%	−6.76%	−3.51%	0%

Table 17 evaluates the influence of customer satisfaction on system performance. The proposed model simultaneously balances profit, quality, cost, and customer utility, resulting in an overall objective value of 0.345. When customer satisfaction is removed from the optimization process, the model focuses primarily on economic objectives, leading to a profit of 1238.524, lower logistics costs, and reduced tardiness cost. However, evaluating the corresponding solution within the proposed framework reveals significant deterioration in customer-related performance measures. Customer satisfaction drops to 0.532 and the fairness value increases to 1.658, indicating a substantial increase in utility inequality among customers. Although product quality increases to 3130.039, the associated allocation decisions do not distribute benefits equitably across customers. Relative to the proposed model, customer satisfaction decreases by 268.42% and fairness deteriorates by 91.68%. These findings confirm that maximizing profit alone is insufficient in cloud manufacturing environments and that explicitly considering customer satisfaction leads to more balanced and sustainable solutions.

**Table 17:** Comparison results between the proposed model and the model without considering customer satisfaction.

Model	Cloud Manufacturing System Profit	Customer Satisfaction	Fairness Among Customers	Overall Objective Function	Total Inter-Factory Logistics Cost	Total Product Delivery Logistics Cost	Product Quality	Total Tardiness Cost	Total Earliness Cost
Proposed Model	1222.191	1.96	0.865	0.345	12.231	74.588	2484.292	316.643	0
Without Considering Customer Satisfaction	1238.524	–	–	–	10.95	63.231	–	301.465	0
Expected Result Without Customer Satisfaction	1240.541	0.532	1.658	0.784	11.524	70.214	3130.039	303.124	0
Improvement Percentage	−1.48%	268.42%	91.68%	127.25%	−5.78%	−5.86%	−20.63%	−4.27%	0%

## 6. Sensitivity analysis

**Fig 4:**
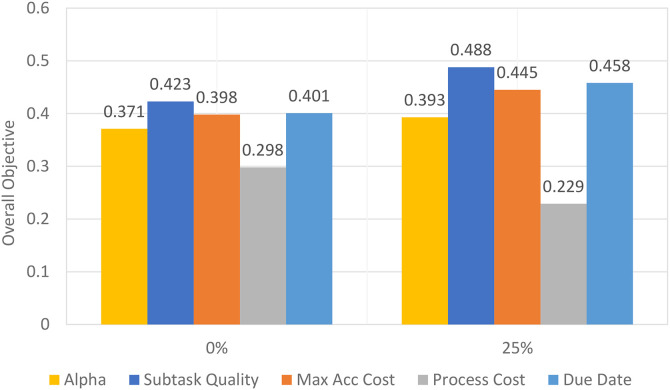
The sensitivity analysis of parametrs on overall objective.

In this section, the sensitivity of the final profit of the cloud manufacturing system (first objective function), customer satisfaction (second objective function), which depends on product price and quality, fairness among customers (third objective function), and the overall objective function is examined with respect to changes in the parameters α, $q_{i}^{f}$, ${MAC}_{j}$, ${PC}_{ij}^{mf}$, and ${DU}_{j}$. For the sensitivity analysis, only one of the parameters is varied at a time while all other parameters remain fixed. The results are presented in Table 18, and Fig 4 illustrate the findings.

**Table 18:** Sensitivity analysis results for α, $q_{i}^{f}$, ${MAC}_{j}$, ${PC}_{ij}^{mf}$ and *DU_j_.*

Parameter	Value	Objective Function Value			
		Cloud Manufacturing System Profit	Customer Satisfaction	Fairness Among Customers	Overall Objective Function
α	0%	1222.191	1.960	0.865	0.345
	25%	1219.842	2.071	0.811	0.371
	50%	1215.936	2.204	0.756	0.393
$q_{i}^{f}$	0%	1222.191	1.960	0.865	0.345
	25%	1233.604	2.296	0.838	0.423
	50%	1248.745	2.688	0.791	0.488
${MAC}_{j}$	0%	1222.191	1.960	0.865	0.345
	25%	1294.851	2.082	0.865	0.398
	50%	1376.413	2.197	0.865	0.445
${PC}_{ij}^{mf}$	0%	1222.191	1.960	0.865	0.345
	25%	1111.286	1.864	0.904	0.298
	50%	992.430	1.732	0.961	0.229
${DU}_{j}$	0%	1222.191	1.960	0.865	0.345
	25%	1308.755	2.038	0.842	0.401
	50%	1398.322	2.146	0.798	0.458

### 6.1. Effect of the minimum utility parameter

The parameter α represents the minimum utility level guaranteed to customers and therefore directly influences the equity mechanism embedded in the proposed optimization framework. As α increases by 25% and 50%, customer satisfaction rises from 1.960 to 2.071 and 2.204, corresponding to improvements of 5.66% and 12.45%, respectively. Simultaneously, the fairness indicator decreases from 0.865 to 0.756. Since lower values indicate smaller deviations among customer utility levels, this result confirms that increasing α substantially improves fairness among customers.

From a modeling perspective, larger values of α tighten the lower bound of customer utility and prevent the optimization model from concentrating resources on only a subset of highly profitable customers. Consequently, the system distributes manufacturing and logistics resources more evenly across customer orders. This redistribution slightly restricts the solution space available for profit maximization, causing profit to decrease marginally from 1222.191 to 1215.936, a reduction of only 0.51%.

The overall objective function increases from 0.345 to 0.393, representing a 13.91% improvement. This finding demonstrates that the gains achieved in customer satisfaction and fairness compensate for the small reduction in profit.

From a managerial perspective, α acts as a strategic fairness-control parameter. Managers seeking long-term customer retention and stronger customer relationships can increase α to guarantee a minimum level of service quality and satisfaction for all customers. The results indicate that substantial improvements in customer equity can be achieved with virtually no sacrifice in profitability.

### 6.2. Effect of factory quality level

Factory quality is one of the most influential operational parameters in the proposed model. Increasing quality by 25% raises customer satisfaction from 1.960 to 2.296, while a 50% increase raises it further to 2.688. These values correspond to improvements of 17.14% and 37.14%, respectively.

The strong sensitivity of customer satisfaction to quality is expected because quality directly enters the customer utility function. Higher manufacturing quality increases the perceived value of products and improves the attractiveness of cloud manufacturing services. In addition, higher quality enables the platform to charge more profitable prices without reducing customer utility.

Profit increases from 1222.191 to 1248.745, representing a growth of approximately 2.17%. Although quality improvements generally require additional operational effort, the resulting increase in customer value generates sufficient additional revenue to offset these costs. The fairness indicator also improves from 0.865 to 0.791 because higher-quality resources become more broadly distributed across customer orders.

The overall objective increases dramatically from 0.345 to 0.488, corresponding to an improvement of 41.45%, which is the largest positive change among all investigated parameters.

From a managerial viewpoint, these findings suggest that investments in advanced manufacturing technologies, process capability improvement, quality-control systems, and workforce development can generate simultaneous benefits for both customers and service providers. Unlike many operational policies that improve one objective at the expense of another, quality enhancement produces a win-win outcome by improving profitability, customer satisfaction, and fairness simultaneously.

### 6.3. Effect of maximum acceptable customer cost

The parameter MACj determines the maximum price customers are willing to accept for manufacturing services. Increasing this parameter effectively expands the feasible pricing region available to the cloud manufacturing platform.

The results show that increasing ${MAC}_{j}$ by 25% raises profit from 1222.191 to 1294.851, while a 50% increase raises profit further to 1376.413. These changes correspond to profit improvements of 5.95% and 12.62%, respectively.

Customer satisfaction also increases moderately from 1.960 to 2.197. Although higher prices generally reduce utility, the optimization model exploits the increased pricing flexibility by selecting higher-quality service combinations that compensate for the increase in customer expenditures. Consequently, customers still experience higher overall utility.

Interestingly, the fairness indicator remains unchanged at 0.865 throughout the analysis. This result suggests that increased willingness to pay affects all customers relatively uniformly and therefore does not alter the distribution of satisfaction among them.

The overall objective improves from 0.345 to 0.445, representing a 28.99% increase.

From a managerial perspective, this finding highlights the strategic importance of customer segmentation. Customers with greater purchasing power can be offered premium service packages involving superior quality, reduced delivery risk, and customized manufacturing solutions. Such differentiated service strategies can significantly increase profitability without compromising customer satisfaction.

### 6.4. Effect of processing cost

Processing cost is the most critical economic parameter in the proposed model. As processing costs increase by 25%, profit decreases from 1222.191 to 1111.286. A 50% increase reduces profit further to 992.430, corresponding to a total reduction of 18.80%.

Because production costs directly influence selling prices, higher processing costs also reduce customer satisfaction. The satisfaction objective declines from 1.960 to 1.732, representing an 11.63% decrease. Customers are therefore required to pay higher prices while receiving similar service quality levels, reducing the attractiveness of the cloud manufacturing platform.

The fairness indicator worsens from 0.865 to 0.961. This result suggests that cost increases do not affect all customers equally. Orders requiring more expensive manufacturing resources experience greater increases in total cost, causing larger disparities in customer utility levels.

The overall objective function decreases sharply from 0.345 to 0.229, corresponding to a decline of 33.62%, which represents the largest negative impact observed in the sensitivity analysis.

From a managerial standpoint, these results emphasize the necessity of cost-control initiatives. Efficient resource utilization, lean manufacturing practices, preventive maintenance programs, automation technologies, and energy-efficiency improvements can significantly enhance overall system performance. Since processing costs simultaneously affect profitability, customer satisfaction, and fairness, controlling these costs should be considered a top managerial priority.

### 6.5. Effect of due date

The due-date parameter governs the scheduling flexibility available to the cloud manufacturing system. Increasing due dates provides additional flexibility for resource allocation and reduces the likelihood of tardiness penalties.

The results indicate that increasing due dates by 25% raises profit from 1222.191 to 1308.755, while a 50% increase raises profit to 1398.322. These values correspond to improvements of 7.08% and 14.41%, respectively.

Customer satisfaction also increases from 1.960 to 2.146 because the optimization model can allocate more suitable manufacturing resources and reduce schedule-related disruptions. At the same time, the fairness indicator improves from 0.865 to 0.798, suggesting that longer due dates allow a more balanced allocation of resources across customers.

The overall objective function increases from 0.345 to 0.458, corresponding to an improvement of 32.75%.

From a modeling perspective, longer due dates enlarge the feasible scheduling region and reduce the pressure imposed by tardiness penalties. Consequently, the model can prioritize quality and resource efficiency rather than focusing exclusively on deadline compliance.

From a managerial perspective, the results demonstrate that realistic delivery commitments play a critical role in cloud manufacturing performance. Extremely tight due dates may force the system to employ expensive resources, increase logistics complexity, and generate substantial tardiness costs. Negotiating feasible delivery schedules can therefore improve profitability, customer satisfaction, and fairness simultaneously.

## 7. Conclusions

Consumers increasingly demand customized products that satisfy their specific requirements, making cloud manufacturing an attractive paradigm for integrating distributed manufacturing resources and services. In response to this challenge, this study proposed a novel multi-objective optimization model that simultaneously integrates scheduling, logistics, pricing, quality, customer satisfaction, and fairness considerations in cloud manufacturing systems. The proposed framework aims to maximize cloud manufacturing system profit, maximize customer satisfaction through a utility-based formulation that incorporates both price and product quality, and minimize inequality in customer utility levels.

The model considers a multi-task environment in which each customer specifies a due date, and earliness and tardiness penalties are explicitly incorporated into the scheduling process. A numerical example was solved using GAMS/CPLEX to demonstrate the applicability of the proposed framework. Comparative analyses showed that neglecting earliness/tardiness, logistics decisions, or customer satisfaction leads to inferior solutions. The results confirmed that logistics integration reduces tardiness and improves coordination among factories, while customer satisfaction considerations encourage higher product quality and more balanced service allocation. Sensitivity analyses further revealed that factory quality and due-date flexibility have the strongest positive effects on overall system performance, whereas processing cost has the most significant negative impact.

To evaluate the scalability of the proposed framework, additional computational experiments were conducted on small-, medium-, and large-scale problem instances. The results showed that the exact CPLEX approach performs efficiently for small instances and remains applicable to medium-sized problems, but its computational requirements increase rapidly as the problem size grows. For large-scale instances, the exact method was unable to obtain optimal solutions within the predefined time limit. Therefore, a genetic algorithm was developed and tested on all benchmark instances. The computational results demonstrated that the genetic algorithm produced solutions very close to the optimal values obtained by CPLEX for solvable instances while significantly reducing computational time for medium- and large-scale problems. These findings confirm the effectiveness of the proposed metaheuristic as a practical solution approach for realistic cloud manufacturing environments.

This study models customer satisfaction from the perspective of industrial service requesters operating in a cloud manufacturing environment. Therefore, satisfaction is represented using operationally measurable criteria rather than subjective consumer perception data. Moreover, the proposed framework assumes a centralized decision-making structure and does not explicitly incorporate customer segmentation strategies, collaborative negotiation mechanisms, or interoperability constraints among distributed manufacturing partners. These aspects may significantly influence decision-making behavior in real-world cloud manufacturing ecosystems and therefore provide promising opportunities for future extensions of the model.

Based on the literature review and identified research gaps, the following suggestions are proposed for future development of the model:

- The cloud manufacturing scheduling model can be extended to consider combined transportation, defined as multiple subtasks with the same origin and destination transported by a single vehicle.
- It is recommended to incorporate setup time/costs into the model to make it more compatible with the practical realities of cloud manufacturin
- Future studies may extend the proposed framework by incorporating customer segmentation strategies based on heterogeneous customer preferences, service priorities, and behavioral characteristics.
- Another promising direction is the integration of collaboration and interoperability mechanisms among distributed cloud manufacturing participants to better reflect real-world decentralized manufacturing ecosystems.
